# PMAIP1, a novel diagnostic and potential therapeutic biomarker in osteoporosis

**DOI:** 10.18632/aging.205553

**Published:** 2024-02-16

**Authors:** Tao Li, Jinghong Yuan, Peichuan Xu, Jingyu Jia, Jiangminghao Zhao, Jian Zhang, Rui Ding, Xiaokun Zhao, Dingwen He, Tianlong Wu, Xigao Cheng

**Affiliations:** 1 Institute of Orthopaedics of Jiangxi Province, Nanchang, Jiangxi, China; 2 Department of Osteoporosis, The Second Affiliated Hospital of Nanchang University, Nanchang, China; 3 Department of Orthopaedics, The Second Affiliated Hospital of Nanchang University, Nanchang, China

**Keywords:** osteoporosis, transcription factors, BMSC, ceRNA, diagnosis

## Abstract

Background: Osteoporosis is a common endocrine metabolic bone disease, which may lead to severe consequences. However, the unknown molecular mechanism of osteoporosis, the observable side effects of present treatments and the inability to fundamentally improve bone metabolism seriously restrict the impact of prevention and treatment. The study aims to identify potential biomarkers from osteoclast progenitors, specifically peripheral blood monocytes on predicting the osteoporotic phenotype.

Methods: Datasets were obtained from Gene Expression Omnibus (GEO). Based on the differentially expressed genes (DEGs) and GSEA results, GO and KEGG analyses were performed using the DAVID database and Metascape database. PPI network, TF network, drug-gene interaction network, and ceRNA network were established to determine the hub genes. Its osteogenesis, migration, and proliferation abilities in bone marrow mesenchymal stem cells (BMSCs) were validated through RT-qPCR, WB, ALP staining, VK staining, wound healing assay, transwell assay, and CCK-8 assay.

Results: A total of 63 significant DEGs were screened. Functional and pathway enrichment analysis discovered that the functions of the significant DEGs (SDEGs) are mainly related to immunity and metal ions. A comprehensive evaluation of all the network analyses, PMAIP1 was defined as osteoporosis’s core gene. This conclusion was further confirmed in clinical cohort data. A series of experiments demonstrated that the PMAIP1 gene can promote the osteogenesis, migration and proliferation of BMSC cells.

Conclusions: All of these outcomes showed a new theoretical basis for further research in the treatment of osteoporosis, and PMAIP1 was identified as a potential biomarker for osteoporosis diagnosis and treatment.

## INTRODUCTION

Osteoporosis (OP), a common clinical metabolic bone disease, is characterized by decreased bone mineral density and degeneration of bone microstructure, resulting in bone fragility [1]. There are more than 200 million OP patients worldwide, with an incidence of more than 25%, ranking sixth in common and frequently-occurring diseases [2]. Osteoporosis is divided into primary and secondary osteoporosis, and primary osteoporosis is divided into postmenopausal osteoporosis (type I), senile osteoporosis (type II) and idiopathic osteoporosis (including adolescent type) [3]. The absolute probability of hip fracture in patients with postmenopausal osteoporosis was less than 1% within five years and did not increase exponentially until the age of 70-79 [4]; senile osteoporosis generally refers to osteoporosis in the elderly after 70 years of age, while idiopathic osteoporosis mainly occurs in adolescents, and the etiology is still unknown [5].

Bone mineral density (BMD) is the gold standard for the diagnosis of OP [6–8], which shows the result of dynamic balance of bone formation and bone resorption. Exploring enhanced bone resorption and decreased bone formation is the focus and difficulty of osteoporosis research, while peripheral blood mononuclear cells (PBMC) can differentiate into osteoclasts and attach and absorb bone on the bone surface [9]. The present study focuses on type I osteoporosis, mainly caused by the deficiency of gonadal function (estrogen and testosterone). Estrogen and testosterone deficiency at any age will accelerate bone mass loss [10–12]. The exact mechanism of bone mass loss is not completely clear, and there are many reasons, mainly the increase of recruitment and sensitivity of pre-osteoclast cells, and the speed of bone resorption is faster than that of bone formation [13, 14].

Estrogen deficiency increases the sensitivity of bone to parathyroid hormone (PTH), resulting in increased calcium loss from bone, decreased renal calcium excretion, and increased production of 25-(OH)2D3 (1,25-dihydroxyvitamin D3) [15–17]. The increase in 1,25-(OH)2D3 promotes calcium absorption in the intestine and kidney and promotes bone resorption by increasing the activity and number of osteoclasts [18, 19]. The secretion of PTH decreases through a negative feedback mechanism, causing the opposite effect [20]. In addition, osteoclasts are also affected by cytokines, such as TNF- α, IL-1 and IL-6, produced by monocytes and increased in the absence of sex hormones [21–24].

Phorbol-12-Myristate-13-Acetate-Induced Protein 1 (PMAIP1) is a pro-apoptotic gene encoding protein of 103 amino acids [25]. This protein, a member of Bcl-2 homology 3 (BH3)–only subtypes from the Bcl-2 family, contains mitochondrial targeting domain (MTD) and BH3 amphipathic helix [26]. The BH3 domain binds the hydrophobic groove of other BCL-2 family members to mediate their interaction [27, 28]. Through this combination, PMAIP1 can neutralise pro-survival proteins Mcl-1 and Bcl2A1 to promote apoptosis [29–31]. Idrus et al. had demonstrated the critical effect of PMAIP1 on osteoclast apoptosis [32]. Due to reduced osteoclast apoptosis, PMAIP1-deficient mice showed an osteoporotic phenotype, such as reduced bone volume fraction and increased osteoclast number. Similarly, chondrocytes lacking the Merlot gene expressed lower levels of pro-apoptotic factors, including PMAIP1, resulting in reduced apoptosis and prolonged lifespan of osteoclasts as well [33].

In the present study, based on GEO datasets, protein and protein interaction network analysis, transcription factors analysis, drug-gene interaction analysis, and ceRNA network analysis were used to screen the potential hub genes PMAIP1 in osteoporosis. Furthermore, we determined they were reliable as a diagnosis of osteoporosis and may be used as a potential therapeutic target for the treatment of osteoporosis.

## MATERIALS AND METHODS

### Patient sets download and processing

In this study, we mainly analyzed mRNA, lncRNA, and miRNA of OP-related PBM. All experimental samples should be extracted from the same tissue to utilize public data for multi-data integration analysis. After searching in Gene Expression Omnibus (GEO; http://www.ncbi.nlm.nih.gov/geo) database, a free public gene expression data repository containing microarray and high-throughput sequencing data, we finally got the appropriate data sets of OP-related PBM for bioinformatics analysis: GSE7158 (mRNA); GSE56815 (mRNA); GSE63446 (miRNA); GSE100609 (lncRNA and mRNA). The original files of CEL format (GSE7158, GSE56815 and GSE63446), offered from GEO databases, were read by affy package (http://www.bioconductor.org/packages/release/bioc/html/affy.html) in R software (3.6.3 version, http://www.R-project.org/) [34]. The read-out microarray data were pretreated and standardized by RMA method. Standardized expression files were re-annotated according to the corresponding annotation platform files, and these re-annotated gene expression matrices of gene symbols were used for further analysis. To analyze the dataset (GSE100609) without the original file from the GEO database, we re-annotated the expression matrix according to the annotation file provided in GEO and obtained the expression matrices of lncRNA and mRNA, respectively. After testing the data dimensions, we utilised log2(exp+1) to standardise the expression matrix to eliminate the data dimensions for subsequent analysis. After that, three mRNA expression matrices, one miRNA expression matrix and one lncRNA expression matrix were obtained for further research.

The Second Affiliated Hospital of Nanchang University provided 48 blood samples of patients with osteoporosis and 48 control specimens from January 2022 to February 2022 as an external validation cohort. All samples were stored at -80° C.

### Differential expression analyses of mRNA, miRNA, lncRNA

The mRNA differential expression analyses were carried out in GSE7158, GSE56815, GSE100609 expression matrices. After the PCA dimensionality reduction analysis, the limma package (http://bioconductor.riken.jp/packages/3.0/bioc/html/limma.html) in R was applied to obtain further the differential expression genes (DEGs) between low-BMD (OP) and high-BMD (Control)) on the preprocessed expression matrices (Selection criteria: P < 0.05) [35]. The miRNA differential expression analyses were carried out in GSE63446. Differential expression miRNAs (DEMs) were acquired with limma package and selection criteria was set at P < 0.05. The lncRNA differential expression analyses were performed with |logFC| > 0.585 and p < 0.05 to control the number of lncRNAs selected. For DEGs, based on the differential expression analyses of the three mRNA expression matrices, the veen tool was used to screen these common DEGs. These outcomes contained together in more or equal two mRNA expression matrix were identified as significant DEGs (SDEGs).

### Functional enrichment analysis of SDEGs

Gene Ontology (GO: https://geneontology.org/) and Kyoto Encyclopedia of Genes and Genomes (KEGG; https://www.genome.jp/kegg/) were included in functional enrichment analyses. In this research, DAVID online tool (https://david.ncifcrf.gov/) and Metascape (https://metascape.org) [36, 37] online tool were used to enrichment analysis for SDEGs.

### Gene set enrichment analysis (GESA) of DEGs

Based on DEGs of GSE56815, which involved the most significant number of samples, Gene set enrichment analysis (GSEA; https://www.gsea-msigdb.org/gsea/index.jsp) was used to clarify the substantial difference in function and pathway between low/high BMD groups [38]. The selected reference gene sets were c2.cp.kegg.v7.3.symbols.gmt (KEGG) and c5.go.v7.3.symbols.gmt (GO), Metric for ranking genes selected t test, other parameters as the software default.

### GSEA of DEMs

For miRNAs, most of their functional predictions are evaluated by the genes they affect. In this study, miEAA (https://ccb-compute2.cs.uni-saarland.de/mieaa2/) was used for GSEA of DEMs [39]. The DEMs are sorted according to logFC. Next, miRNA matures were obtained by ID conversion using miEAA according to the miRNA precursors. Furthermore, GSEA of miRNA was also performed through miEAA. The enrichment analysis of differential miRNA by miEAA was performed towards GO and KEGG.

### Protein-protein interaction (PPI) network construction and analysis

GeneMANIA (http://genemania.org/searc) [40], a tool that can be used to identify related genes, including protein-protein, protein-DNA and genetic interactions, pathways, physiological and biochemical reactions, gene and protein expression, protein domains and phenotypic screening, and the data are updated regularly, was used to construct and analyse the PPI network of SDEGs. SDEGs were uploaded to GeneMANIA to get the interactions between SDEGs and more potentially associated proteins and visualised using Cytoscape software (version 3.7.2, https://cytoscape.org/) [41].

MCODE is a plug-in of Cytoscape software, which uses the Vertex-Weighting scheme to find the locally high-density area in the graph. According to the default parameters MCODE was used to determine the crucial gene clusters in the PPI network. The sub-network obtained by MCODE were identified as hub genes, which could be used as molecular targets for further experiment.

### Transcription factors network construction

IRegulon [42], a plug-in to Cytoscape, was used to predict the transcription factors in regulating gene sets through motif enrichment analysis in the present study. Multiple position weight matrices (PWM) were used to rank each motif in motif enrichment analysis, and then the preferred motif was used to determine the transcription factors further. The plug-in integrates the transcription factors of target gene predicted by motif in multiple transcription factor databases, such as TRANSFAC, JASPAR, ENCODE, SwissRegulon, HomeRanD, etc. It can also predict the TF-target gene network and TF-miRNA network. The predictable species are humans, mice and flies. In addition, “iRegulon” can also provide a query for human TF target genes from MSigDB, GeneSigDB, and Ganesh Clusters. Default parameters were chosen to predict and analyze the transcriptional factors from SDEGs. In addition, the prediction results were visualized by Cytoscape software.

### Drug target network construction of SDEGs

DGIdb database (Drug-Gene Interaction database) is a database of drug-gene interactions which provides information about the association between genes and their known or potential drugs [43]. The genes are mainly oncogenes but also include some genes related to other diseases (such as Alzheimer’s, heart disease, diabetes, etc.). DGIdb has more than 14,000 drug-gene interactions, involving 2,600 genes and 6,300 drugs that target these genes, as well as 6,700 other genes, which are likely to become future drug targets. After uploaded SDEGs on DGIdb, FDA approved as Preset Filters, the drug-gene interaction analysis was determined, and these results were visualized to a network using Cytoscape software.

### Competing endogenous RNA (ceRNA) network construction

MiRWalk 2.0 (http://zmf.umm.uni-heidelberg.de/apps/zmf/mirwalk2/) not only records the miRNA binding sites on the full-length sequence of genes [35] but also associates them with the predicting binding information sets of 12 existing miRNA target prediction programs (DIANA-microTv4.0, DIANA-microT-CDS, miRanda-rel2010, mirBridge, miRDB 4.0, miRmap, miRNAMap, doRiNA, PicTar 2, PITA RNA22 v2, RNAhybrid 2.1 and Targetscan 6.2), aiming at establishing a brand-new alignment platform, including promoter (four prediction databases), CDS region (five prediction databases), 5’-UTR region (five prediction databases) and 3’-UTR region.

DEMs were uploaded on miRWalk to predict their target mRNAs. Among the 12 databases, at least more than six databases can predict miRNA-targeted mRNA, which can be regarded as a reliable miRNA-mRNA interaction. Then, we extracted the regulatory miRNA-mRNA corresponding to SDEGs from all the reliable prediction results and determined the miRNA-mRNA regulatory network used to construct the ceRNA network. Afterwards, we performed lncRNA-related prediction analysis on differential miRNAs by five related databases/methods (miRWalk, miRanda, PITA, RNAhybrid, Targetscan), and other parameters are default. Results supported by at least more than two databases/methods in all the prediction results were taken as reliable lncRNA-miRNA prediction results obtained in this analysis. Then, we extracted the regulatory miRNA corresponding to DELs from all the reliable prediction results and got the lncRNA-miRNA regulatory network used to establish the ceRNA network further. Based on Cytoscape software, the reliable miRNA-mRNA and miRNA-lncRNA interactions were visualized as an OP-PBM-related ceRNA network.

### Cell culture and cell transfection

BMSC were cultured in a complete medium, including 10% fetal bovine serum (Gibco, USA), 1% penicillin-streptomycin solution (NCM Biotech, China) and 89% DMEM incomplete medium (Gibco, USA). Small interfering RNAs (siRNAs) for PMAIP1 and their negative control (NC) were obtained from RiboBio (Guangzhou, China). Cell transfection was performed when the cell density reached 50%-60%.

### RT-qPCR

PBMC were isolated from heparinized venous blood using Ficoll sodium diatrizoate gradient centrifugation (Sigma-Aldrich, St. Louis, MO) and were lysed in TRIzol® reagent (Thermo Fisher Scientific, Inc.). Using the RNA Extraction (G3013, Servicebio), the total RNA was extracted and stored at −80° C. Expression levels were detected using Servicebio®RT First Strand cDNA Synthesis Kit (G3330, Servicebio) and SYBR Green qPCR Master Mix (G3320, Sevicebio). The temperature protocol for reverse transcription was 25° C for 5 min, 42° C for 30 min and 85° C for 5 sec. And cDNA was subjected to initial denaturation at 95° C for 10 min, followed by 40 cycles at 95° C for 15 sec and 60° C for 30 sec, followed by extension from 65° C to 95° C and the fluorescence signal was collected once every 0.5° C temperature rise, using the specific primers. All the primers were purchased by Servicebio, China. All experiments were repeated three times. Primer sequences were presented in Supplementary Table 1. The 2-ΔΔCq method was used for relative quantification.

### Western blotting analysis

Total proteins were extracted from BMSC cells using RIPA buffer, and then 6X loading buffer was added. Protein concentration was measured using the BCA method and adjusted to the same concentration. SDS-PAGE was performed on the 10% polyacrylamide gel, and the protein samples were transferred onto PVDF nitrocellulose membranes (Millipore, Bedford, MA). The PVDF membranes were blocked with 5% non-fat milk for 90 minutes, washed twice with PBS, and then incubated overnight at 4° C with primary antibodies, including GAPDH, OPN, OCN, and RUNX2 (Proteintech, China). After washing twice with PBS, the PVDF membranes were incubated with secondary antibodies at room temperature for 2 hours. Finally, the proteins were analyzed using a fluorescent imaging analyser. The experiments were repeated three times, and the data were analyzed using ImageJ software.

### Alkaline phosphatase (ALP) staining

Alkaline phosphatase staining was determined using the alkaline phosphatase assay kit according to the manufacturer’s protocol (Servicebio, China). The results were imaged under a microscope (Olympus). The ALP staining intensity was quantified using the ImageJ software.

### Von kossa(VK) staining

Von kossa staining was determined using the von kossa assay kit according the manufacturer’s protocol (Servicebio, China). BMSCs were added with von kossa staining solution, irradiated with ultraviolet light for 4 hours, washed with ultra-pure water, and re-dyed with hematoxylin eosin. The results were imaged under a microscope (Olympus). The von kossa staining intensity was quantified using the ImageJ software.

### Transwell assay of migration

Transwell chambers that have been washed with alcohol and air-dried were placed in a 24-well plate. 2x10^5 cells in 100 uL of serum-free DMEM were seeded in the top compartment, while 0.6 mL of DMEM containing 10% fetal bovine serum was added to the bottom compartment. The 24-well plate was incubated at 37° C for 24 hours. Then, the transwell chambers were removed, washed twice with PBS, and fixed with paraformaldehyde. The cells were stained with crystal violet and counted under a microscope.

### Wound healing assay

BMSC cells were seeded into a 6-well plate and allowed to grow to a density of approximately 90%. A sterile pipette tip (200μl) created a scratch in the adherent cells. The cells that detached during the scratch were washed away with PBS solution, and a fresh complete medium was replaced. The degree of wound healing was calculated by taking microscopic images of the scratch at 0 and 24 hours.

### CCK-8 assay

To determine the cell proliferation ability, we performed a CCK-8 assay. The treated NC blank group and Si-PMAIP1 group cells were separately seeded into a 96-well plate, with 100 μL of cell suspension added to each well. Five replicate wells were set for each group. After incubating for 24 hours until the cells adhered to the plate, we added ten μL of CCK-8 reagent and incubated for 3 hours. Then, we measured the OD value at 450 nm and analyzed the results statistically.

### Statistics analysis

In this research, the diagnostic accuracy of PMAIP1 areas under curve (AUC) of receiver operating characteristic (ROC) analysis using pROC package (1.17.0.1 version) and visualized by ggplot2 package (3.3.5 version) in R. R statistical software (3.6.3 Version) and Excel (Microsoft office 2019) were used to statistical analysis. The Student’s t-test was used to test the statistical significance of differences between the two groups. *, p-value < 0.05; **, p-value < 0.01; ***, p-value < 0.001; ****, p-value < 0.0001.

### Availability of data and material

The datasets analysed for this research can be found in the Gene Expression Omnibus (GEO; GSE7158, https://www.ncbi.nlm.nih.gov/geo/query/acc.cgi?acc=GSE7158;

GSE56815, https://www.ncbi.nlm.nih.gov/geo/query/acc.cgi?acc=GSE56815;

GSE63446, https://www.ncbi.nlm.nih.gov/geo/query/acc.cgi?acc=GSE63446;

GSE100609, https://www.ncbi.nlm.nih.gov/geo/query/acc.cgi?acc=GSE100609).

## RESULTS

### Screening of DEGs

**Figure 1:**
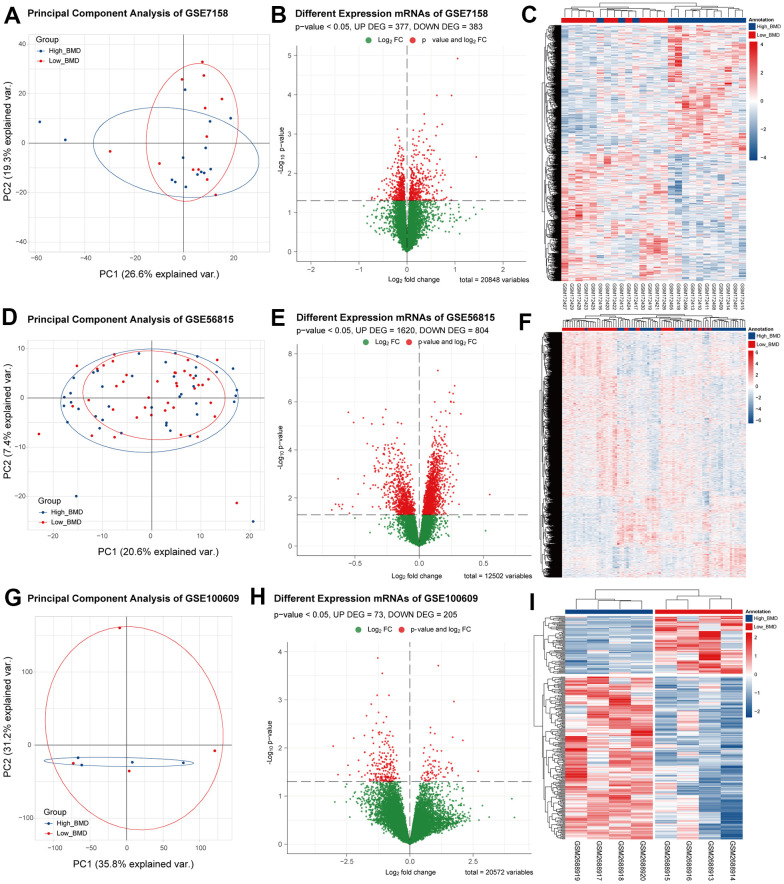
**The difference analysis results of the three datasets.** (**A**–**C**) PCA dimensionality reduction analysis, difference analysis volcano map and bidirectional clustering heat map of differential genes for GSE7158. (**D**–**F**) PCA dimensionality reduction analysis, difference analysis volcano map and bidirectional clustering heat map of differential genes for GSE56815. (**G**–**I**) PCA dimensionality reduction analysis, difference analysis volcano map and bidirectional clustering heat map of differential genes for GSE100609.

According to the method section, the limma package in R was used to conduct difference analysis on the three mRNA expression matrices, respectively, and a P-value < 0.05 was set as the screening threshold of DEGs. The difference analysis results were shown using PCA dimensionality reduction analysis charts, volcano maps, and heat maps (Figure 1).

### Identifying significant DEGs (SDEGs) and functional enrichment analysis

**Figure 2:**
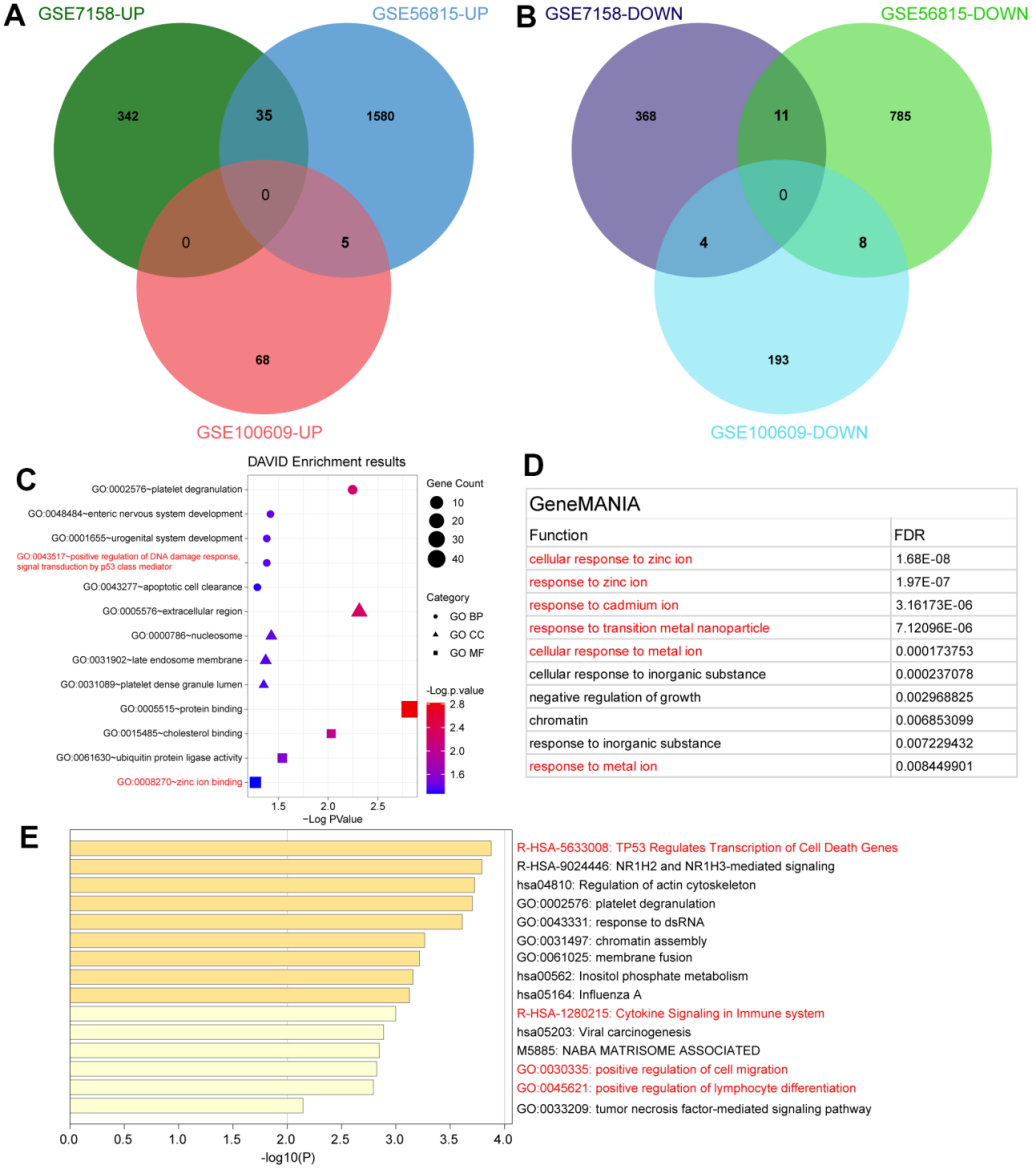
**Identification and enrichment analysis of SDEGs.** (**A**) Up-regulated SDEGs in the three datasets. (**B**) Down-regulated SDEGs in the three datasets. (**C**) Significant functional enrichment analysis results of SDEGs using the DAVID tool. (**D**) Significant functional enrichment analysis results of SDEGs using GeneMANIA database. (**E**) Significant functional enrichment analysis results of SDEGs using Metascape database.

To further identify significant DEGs (SDEGs), the jveen tool was used to screen these crucial SDEGs. These outcomes revealed that 40 up-regulated and 23 down-regulated SDEGs were determined (Figure 2A, 2B). According to DAVID online tool enrichment analysis, these results showed that SDEGs enrichment terms included “positive regulation of DNA damage response signal transduction by p53 class mediator” and “zinc ion binding” (Figure 2C). Based on outcomes of GeneMANIA, SDEGs were mainly enriched in “cellular response to zinc ion”, “response to zinc ion”, “response to cadmium ion”, “response to transition metal nanoparticle”, “cellular response to metal ion”, and “response to metal ion” (Figure 2D). In addition, these results of Metascape tool analysis showed that SDEGs were enriched in “TP53 Regulates Transcription of Cell Death Genes”, “Cytokine Signaling in Immune system”, “positive regulation of cell migration”, and “positive regulation of lymphocyte differentiation” (Figure 2E).

### PPI network analysis

**Figure 3:**
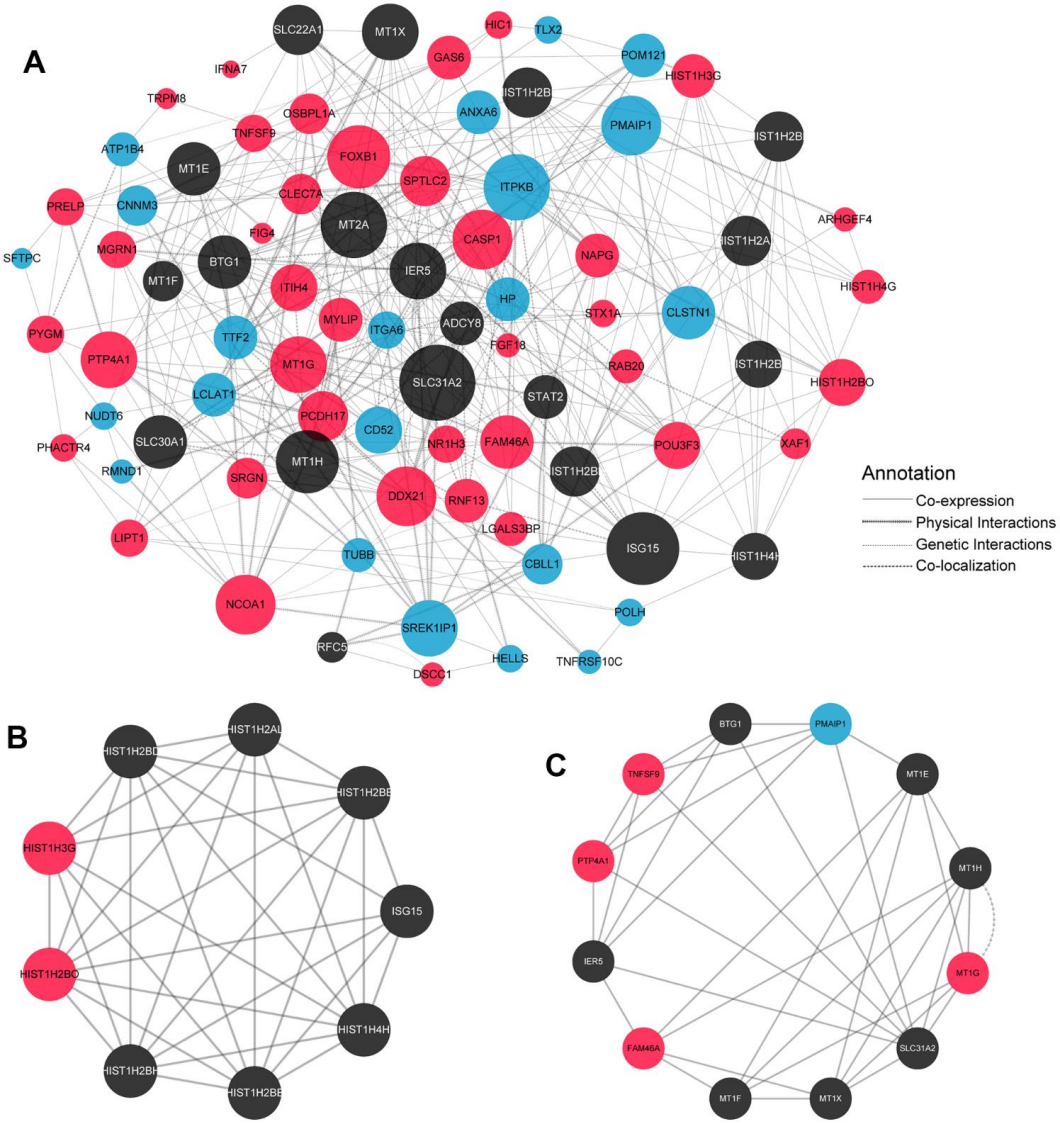
**PPI network of SDEGs and subnetwork.** (**A**) PPI network construction of differential genes in GeneMANIA database. Red dots: significant up-regulate differential genes; Blue dots: significant down-regulate differential genes; Black dots: related genes added by the GeneMANIA database for the associate PPI network. Circle size indicated the degree of the corresponding gene in the PPI network. The larger the circle is, the greater degree of the corresponding node in the figure, which can explain why this node is more important in the network from the point of view of graph theory. (**B**, **C**) Utilising the MCODE plug-in to analyse the PPI network, two key subnetworks were obtained (Figure 3B: the subnetwork of Score8.5; Figure 3C: the subnetwork of Score6.182.).

To further clarify the interaction and regulation between SDEGs, the PPI network was constructed based on the GeneMANIA database (Figure 3A). Furthermore, crucial gene clusters were selected using the MCODE clustering algorithm. Based on these outcomes, two key gene clusters were determined (Figure 3B, 3C). After the non-SDEGs added by GeneMANIA to establish the PPI network removed, six up-regulated SDEGs (HIST1H3G, HIST1H2BO, PTP4A1, FAM46A, MT1G and TNFSF9) and one down-regulated SDEG (PMAIP1) were identified as potential hub genes in the pathogenesis of osteoporosis. They may become potential targets for the treatment of osteoporosis.

### Transcription factors analysis

**Figure 4:**
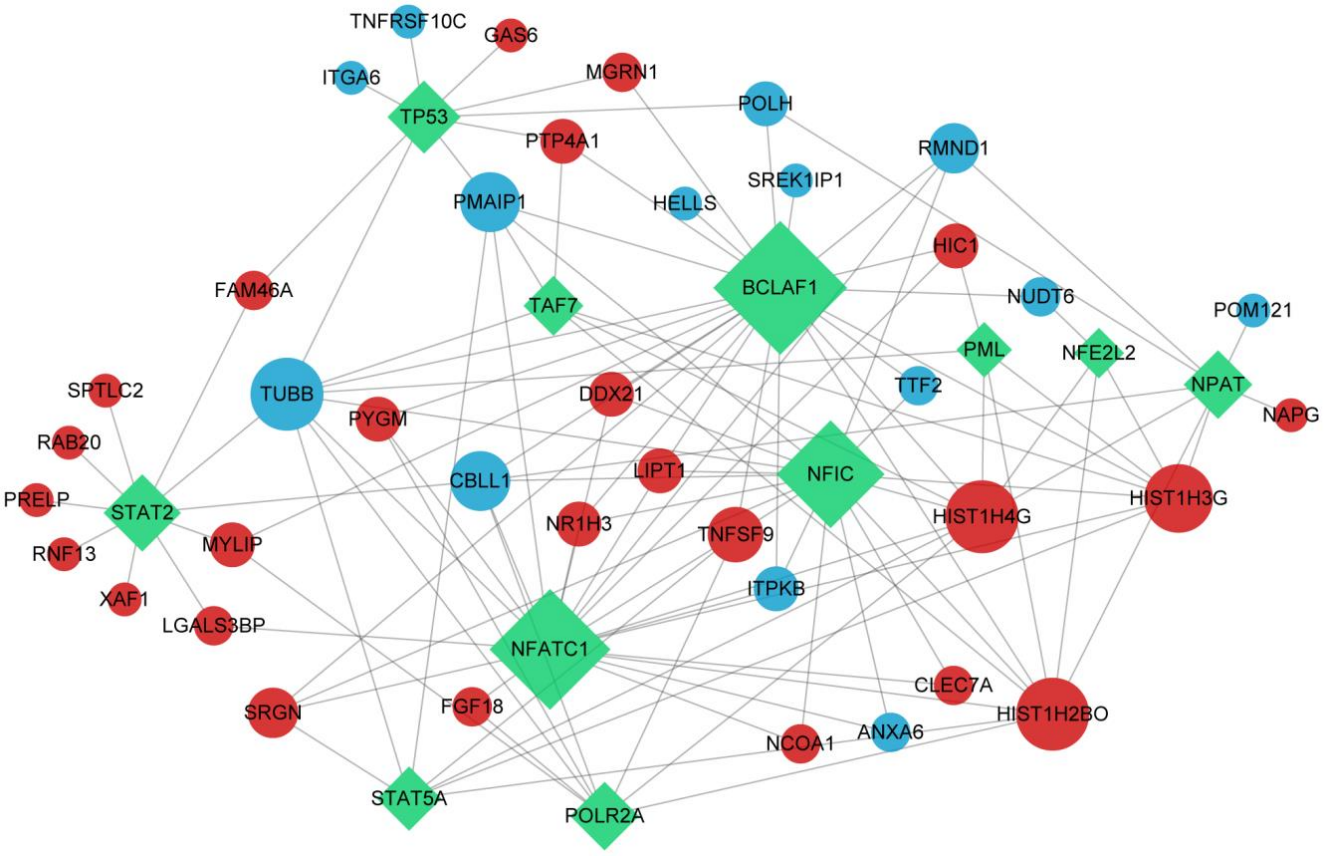
**Transcription factors network.** The green rectangle in the graph represents transcription factors; red and blue dots indicate up-regulated and down-regulated significant differential gene, respectively.

According to the method section, we predicted the potential mRNA and transcription factors interactions of SDEGs. These results were performed as a transcription factors network and visualized using Cytoscape software (Figure 4), including 11 transcription factors (Green), 14 down-regulated SDEGs (Blue) and 25 up-regulated SDEGs (Red).

### Drug targets analysis

**Figure 5:**
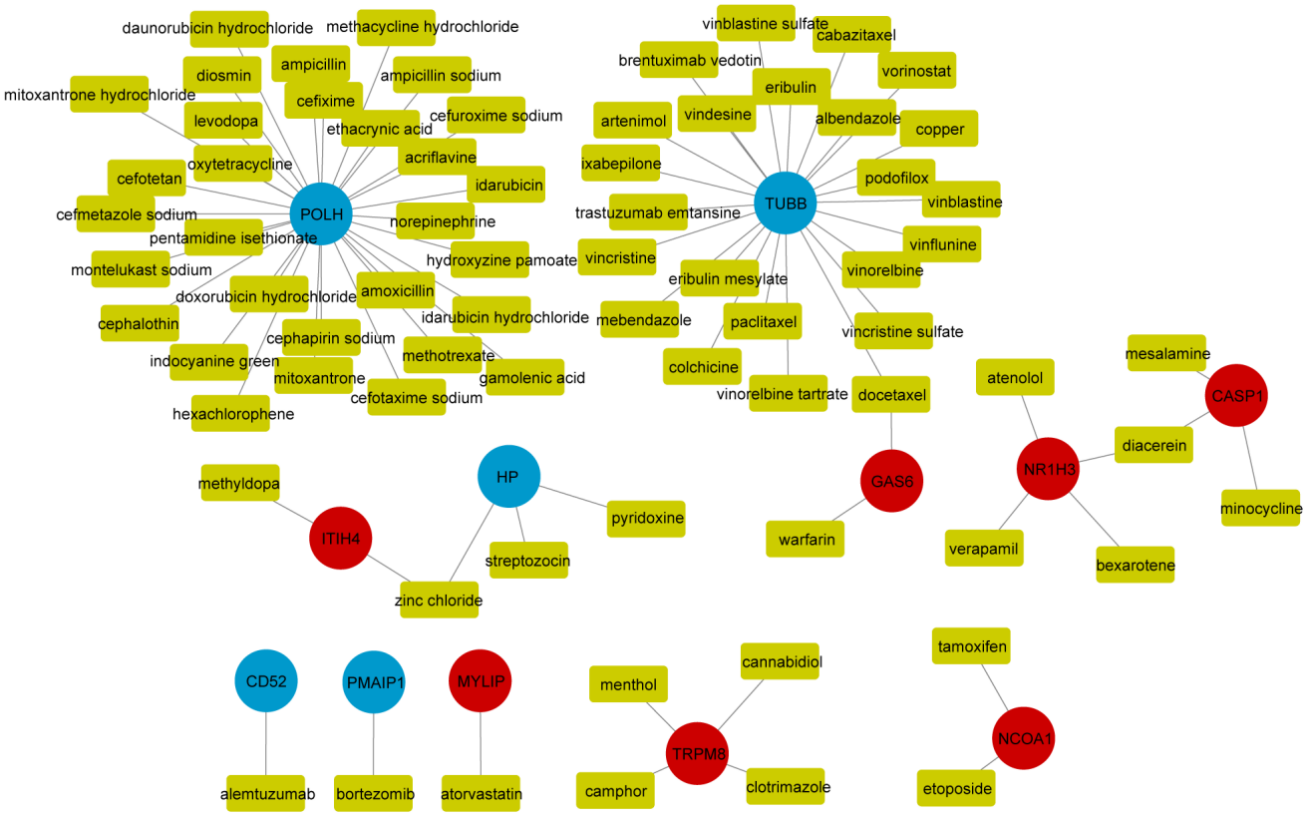
**Drug-mRNAs network.** The yellow rectangle in the graph represents the drugs predicted by the DGIdb database; red and blue dots indicate up-regulated and down-regulated significant differential genes, respectively.

Based on the DGIdb database, a drug-mRNA network was determined and visualized by Cytoscape software (Figure 5), including 64 predicted drugs (Yellow), five down-regulated SDEGs (Blue) and seven up-regulated SDEGs (Red).

### DEMs and DELs screened, and GSEA

**Figure 6:**
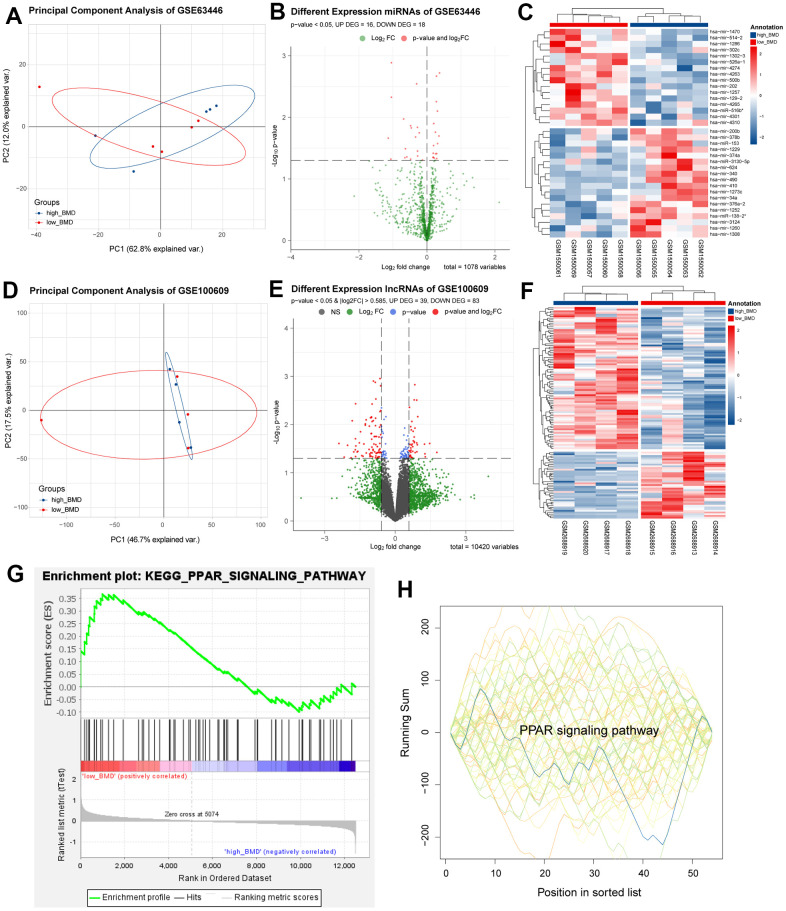
**Differentially expressed mRNA and lncRNA analysis.** (**A**–**C**) Differentially expressed miRNA. (**D**–**F**) Differentially expressed lncRNA. (**G**, **H**) Common pathways obtained by GSEA analysis of mRNA and miRNA, respectively.

Similarly, the limma package in R was applied to screen DEMs (16 up-regulated and 18 down-regulated) and DELs (39 up-regulated and 83 down-regulated). Then, these outcomes were plotted in Figure 6A–6F. Moreover, GSEA analysis was performed between the DEGs of GSE56815, with the largest sample size set and DEMs of GSE63446. The GSEA analysis was performed towards GO and KEGG. These results revealed that the PPAR signaling pathway was obtained from the GSEA KEGG enrichment results of these two datasets and suggested that the PPAR signaling pathway may exert essential roles in the progression of osteoporosis. PPAR signaling pathway-related proteins and their interactions were shown in Supplementary Figure 1.

### CeRNA network analysis

**Figure 7:**
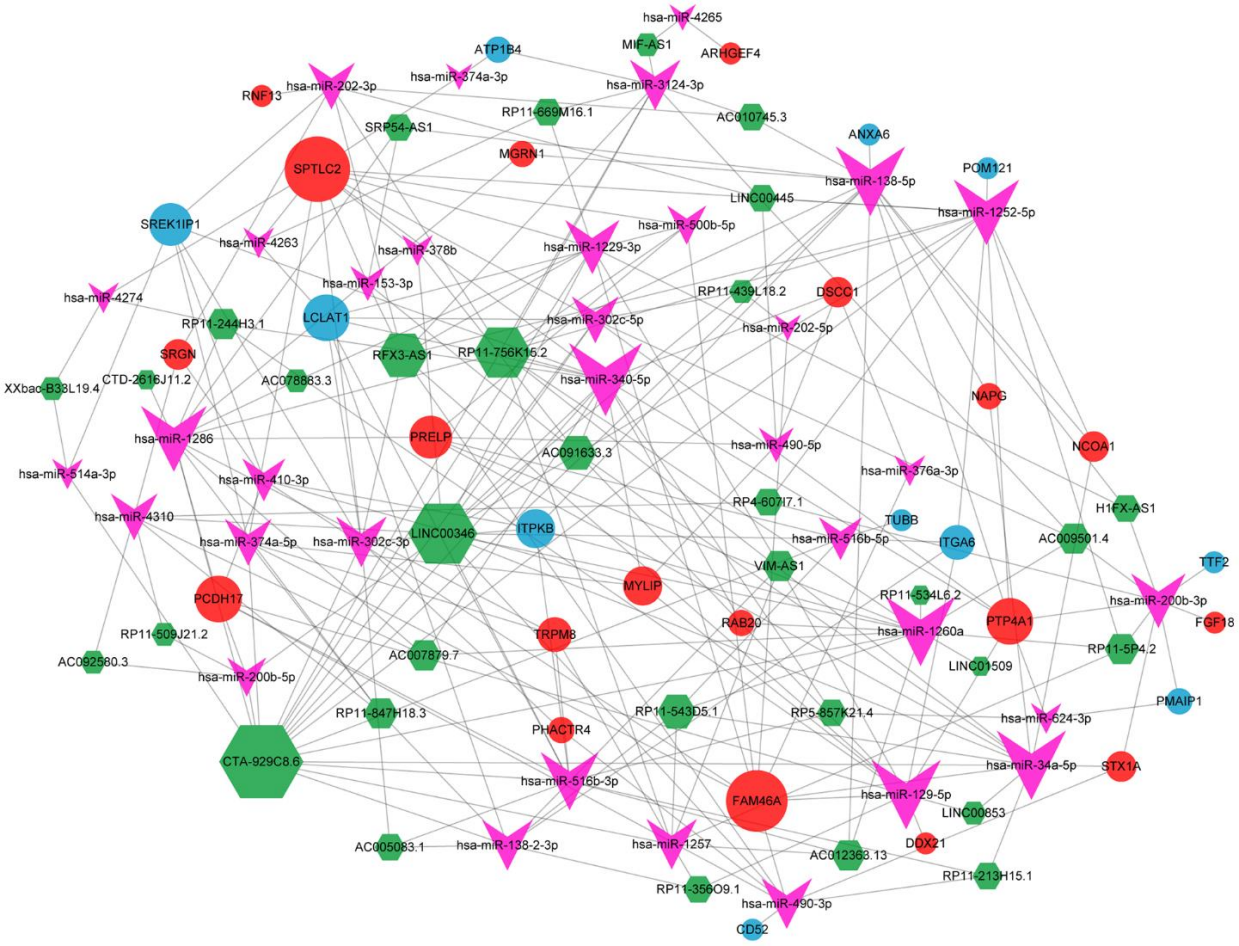
**CeRNA network.** Red and blue dots indicated up-regulated and down-regulated significant differential genes, respectively. The green hexagon refers to significant differential lncRNA, and the pink arrow is a significant differential miRNA; the size of the shape indicates the degree size of the corresponding node in the network, and the larger the shape, the larger the corresponding node in the network, the more influential the node is in the ceRNA network.

The ceRNA interaction relationship was obtained by the above method, and the ceRNA network was constructed and visualized using Cytoscape software, including 33 SDEGs (19 up-regulated and 11 down-regulated), 33 DELs and 34 DEMs, with a total of 253 edges (Figure 7).

### Identification of the key gene

**Figure 8:**
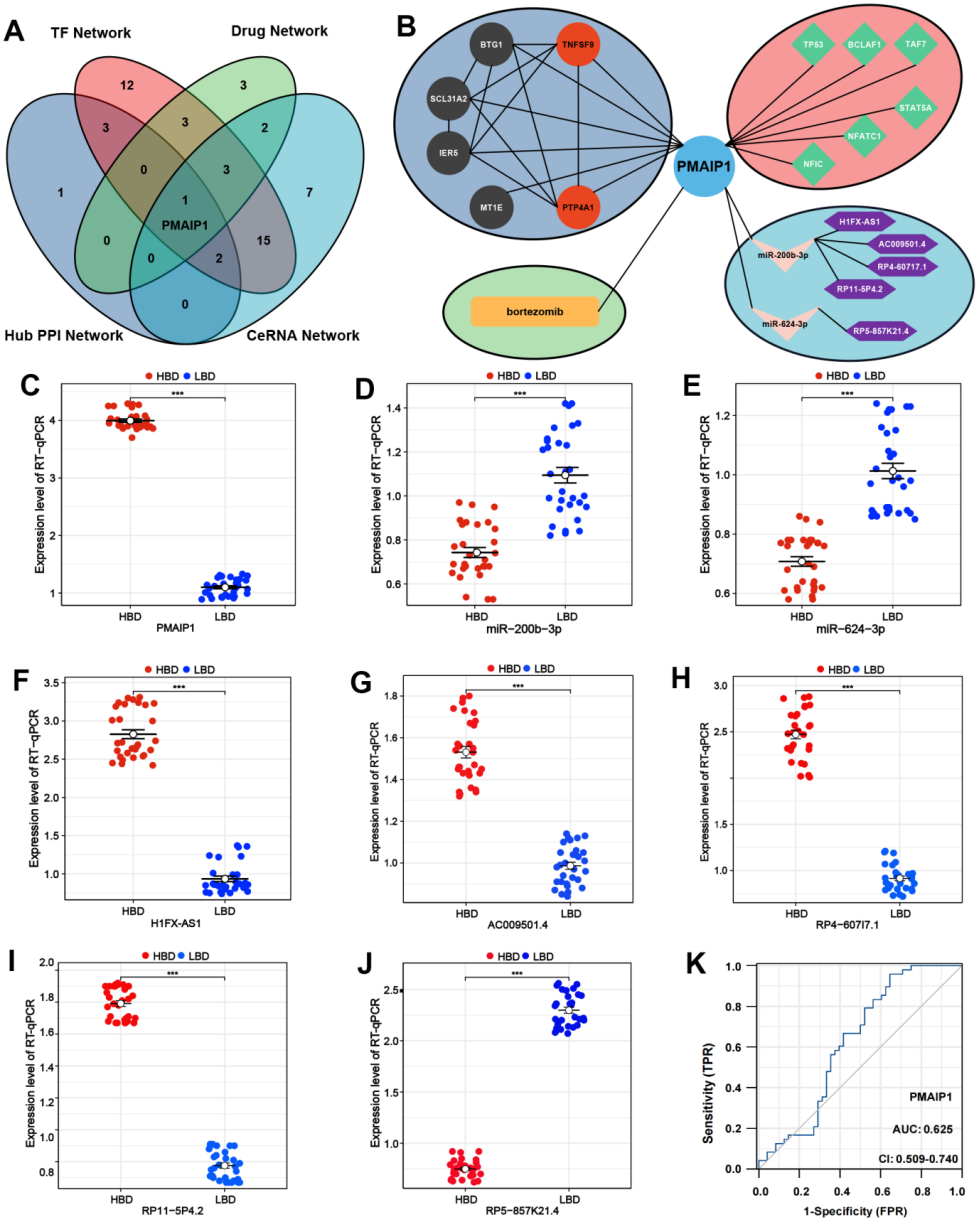
**Crucial genes validation and diagnostic model construction of ISS.** (**A**) Venn diagram of intersected genes of hub PPI network, TF network, Drug-mRNA network, and ceRNA network. (**B**) Network analysis of PMAIP1. (**C**–**J**) The expression levels of PMAIP1 and PMAIP1-related genes. (**K**) Receiver operating characteristic (ROC) for predictive values of PMAIP1.

Based on PPI network analysis, TF network analysis, drug targets network analysis, and ceRNA network analysis, a Venn plot revealed that PMAIP1 may be the crucial gene in osteoporosis (Figure 8A), and PMAIP1-related network was shown in Figure 8B. The expression levels of PMAIP1 and PMAIP1-related in the ceRNA network (miR-200-3p, miR-624-3p, H1FX-AS1, AC009501.4, RP11-5P4.2, RP4-607I7.1, RP5-857K21.4) were evaluated by RT-qPCR and the results showed that PMAIP1 demonstrated the most significant difference (Figure 8C–8J). Then, the diagnostic ROC curves outcomes showed that PMAIP1 can be used as the diagnostic biomarker (Figure 8K).

### Validation of the influence of PMAIP1 on osteogenesis, migration and cell growth of BMSCs

**Figure 9:**
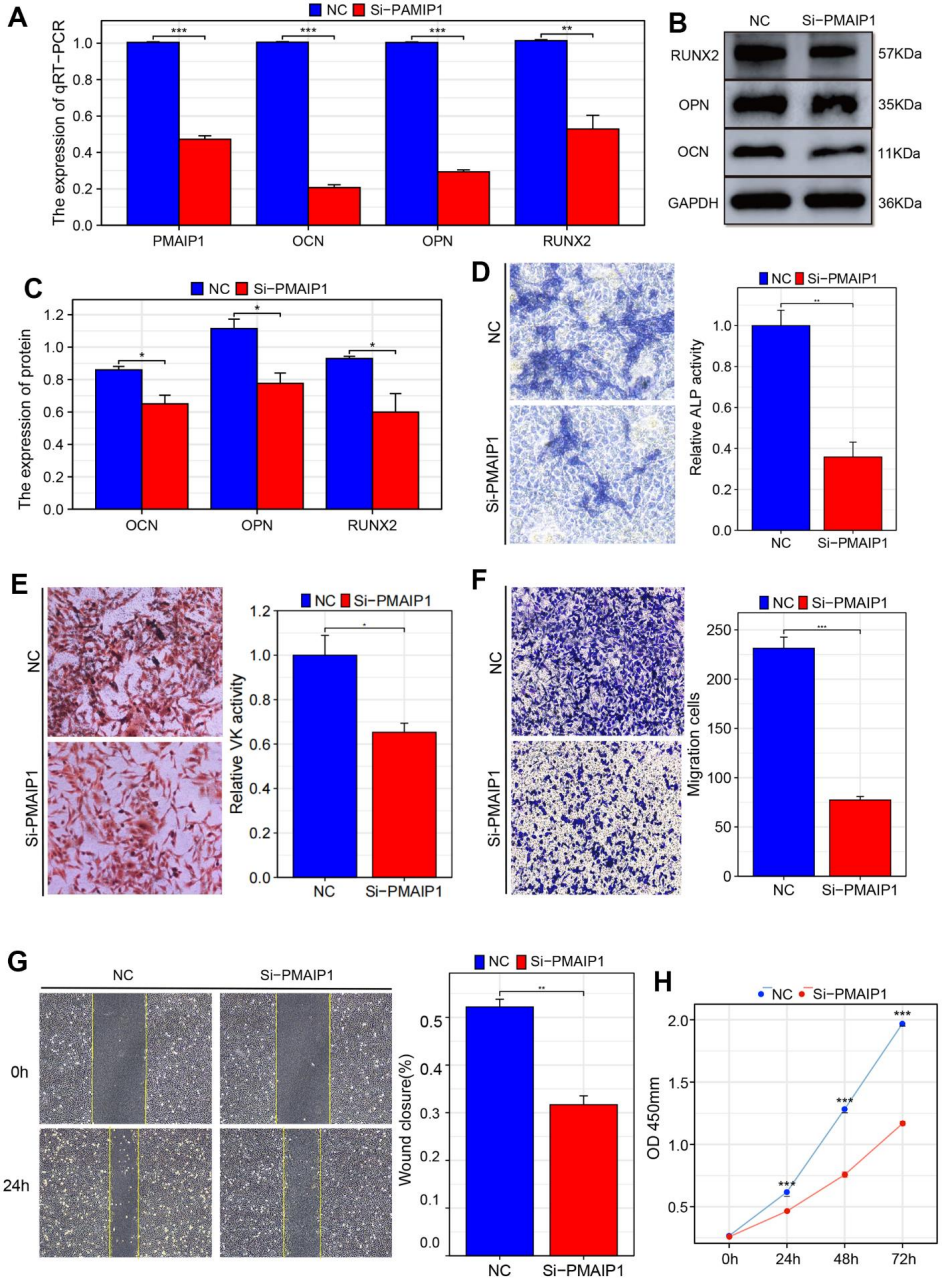
**Validation of the influence of PMAIP1 on osteogenesis, migration, and cell growth of BMSCs.** (**A**) RT-qPCR (**B**, **C**) Western blotting (**D**) ALP staining (**E**) VK staining (**F**) Transwell assay of migration (**G**) Wound healing assay (**H**) CCK-8 assay.

Firstly, PMAIP-1 was successfully knocked down, and this result was validated by RT-qPCR (Figure 9A). RT-qPCR results showed that compared to the standard control group, osteogenic markers RUNX2, OPN and OCN expression levels were significantly reduced in the PMAIP1 knockdown group (Figure 9A). This result was further confirmed in the Western blot experiment (Figure 9B, 9C). The ALP and VK staining showed a significant decreased osteogenesis of the BMSC cells in the Si-PMAIP1 group. In addition, transwell assay and wound healing were performed, and the knockdown of PMAIP1 resulted in decreased migration in BMSC cells (Figure 9F). Then, the CCK-8 assay was performed, and the cell proliferation was inhibited after knocking down the gene (Figure 9G).

## DISCUSSION

OP is a progressive systemic skeletal disease that causes up to 40% risk of lifelong fractures [44–46]. With the intensification of the ageing process, the incidence of OP is increasing, which puts a substantial economic burden on the health system [47]. Thus, advances in the molecular mechanism of OP are helpful for us to identify diagnostic biomarkers and develop new therapeutic targets. In the present study, bioinformatic analysis was performed to explore the key genes and pathways predict TF-mRNA and drug-mRNA network of OP based on four mRNA expression matrices from GEO. 63 SDEGs (40 up-regulated and 23 down-regulated) were screened. Functional and pathway enrichment analysis revealed that the SDEGs mainly function in immunity and response to metal ions. More than 99% of the body’s calcium storage is in the skeleton in the form of hydroxyapatite, which provides bone strength and acts as a calcium pool to maintain the dynamic balance of blood calcium [48, 49]. High extracellular Ca^2+^ concentration can lead to changes in the cytoskeleton and function of osteoclasts, thus reducing bone resorption [50–52]. Besides, it can also promote the proliferation, differentiation and migration of osteoblasts [53, 54]. As an essential nutrient element, zinc forms many enzymes in the body [55, 56]. Zinc has a positive effect on fracture healing [57, 58]. It stimulates the proliferation and differentiation of osteoblasts and inhibits the bone resorption of osteoclasts [59–61]. There is a significant increase in urinary zinc excretion in postmenopausal patients with osteoporosis, which indicates its potential as a marker of bone resorption [62]. The related functions of SDEGs in immunity and response to metal ions further suggest that the changes in these molecular functions of monocytes may be closely associated with osteoporosis.

After PPI network analysis, 6 up-regulated SDEGs (HIST1H3G, HIST1H2BO, PTP4A1, FAM46A, MT1G and TNFSF9) and one down-regulated SDEG (PMAIP1) were determined as hub genes. Then, TFs are reserved for proteins binding to DNA sequence-specifically ornon-DNA-binding accessory proteins, which can regulate gene transcription and cellular functions [63–67]. In this research, 11 transcription factors (TP53, BCLAF1, STAT5A, STAT2, NFATC1, POLR2A, NFIC, PML, NFE2L2, TAF7 and NPAT), 14 down-regulated SDEGs (TNFRSF10C, IGTA6, PMAIP1, HELLS, POLH, SPEK1IP1, RMND1, NUDT6, POM121, TTF2, ITPKB, ANXA6, CBLL1 and TUBB) and 25 up-regulated SDEGs (GAS6, MGRN1, PTP4A1, HIC1, FAM46A, SPTLC2, RAB20, PRELP, RNF13, XAF1, MYLIP, LGALS3BP, SRGN, PYGM, FGF18, DDX21, NR1H3, LIPT1, TNFSF9, NCOA1, CLEC7A, HIST1H4G, HIST1H3G, HIST1H2BO and NAPG) were included in a TFs-mRNAs network. Plenty of TFs act as significant parts of OP pathogenesis [68]. As to osteoclast regulation, pleiotropic TF, nuclear factor kappa-B (NFκB), is effective in osteoclast formation, function, and survival [69]. Yamashita et al. [70] found that TFs c-Fos and NFATc1 were activated via NF-κB signalling, accelerating osteoclast differentiation. On the contrary, the overexpression of Lhx2 in osteoclast precursor cells inhibited osteoclast differentiation by inhibiting the binding of c-Fos to NFATc1 promoters [71]. In osteoporosis, bone marrow stromal cells (BMSCs) differentiate less into bone and more into fat [72, 73]. In addition, seven up-regulated SDEGs (ITIH4, MYLIP, TRPM8, GAS6, NR1H3, NCOA1 and CASP1), five down-regulated SDEGs (POLH, TUBB, HP, CD52 and PMAIP1) and 63 kinds of drugs were included in the drug-mRNAs network. These outcomes provided a potential basis for elucidating novel mechanisms of osteoporosis and finding novel therapeutic targets for osteoporosis.

In recent studies, many researchers have discovered that non-coding RNAs (circRNA, lncRNA, and microRNA) are significant in the underlying mechanism and role of osteoporosis [74–77]. To further clarify the role of non-coding RNAs in OP, we determined 34 DEMs (16 up-regulated and 18 down-regulated) from GSE63446. The results of GSEA between DEGs of GSE56815 and DEMs of GSE53446 revealed that the common enrichment pathway, the PPAR signalling pathway, was recognised as an important role in the occurrence and development of osteoporosis. PPARs are ligand-inducible nuclear receptors that control many intracellular metabolic processes [78, 79]. At present, three subtypes of PPARs: PPARα, PPARβ/δ, and PPARγ, have been identified in mammals [21]. The role of PPARs in bone metabolism had received a wide range of research. Both osteoblasts and osteoclasts can be adjusted by PPARγ. PPARγ regulates C-Fos directly to increase osteoblasts, and the lack of PPARγ stimulates osteoblasts’ differentiation to increase bone mass [80, 81]. In contrast, PPARβ/δ can increase the expression of osteoprotegerin by activating the Wnt signal pathway, resulting in reduced osteoclast formation mediated by osteoblasts [82]. Although no studies prove the effect of PPARα on bone balance, Kim et al. found that PPARα agonist fenofibrate increased PPARα and bone morphogenetic protein 2 in dose and time to enhance osteoblast differentiation [83].

Furthermore, 122 DELs (39 up-regulated and 83 down-regulated) were selected from GSE100609. Then, we constructed the ceRNA network, including 34 miRNAs, 33 lncRNAs and 30 mRNAs (19 up-regulated, 11 down-regulated). Comprehensive evaluation of the PPI network, TFs network, drug targets network, ceRNA network analysis results, PMAIP1 were defined as the core genes of osteoporosis. PMAIP1, belongs to pro-apoptotic subfamily within the BCL-2 protein family, referred to as the BCL-2 homology domain 3 (BH3)-only subfamily, which regulates apoptosis and proliferation of various tumor cells [84–87]. In terms of bone metabolism, PMAIP1 knockout mice showed decreased osteoclastogenesis and increased osteoclast number [32]. To further investigate the role of PMAIP1 in osteoporosis, RT-qPCR confirmed that PMAIP1 was the most differentially expressed gene. Additionally, the ROC curve analysis result was meaningful, demonstrating that PMAIP1 can serve as a diagnostic molecular marker for osteoporosis diagnosing.

Then, this gene’s biological function was identified through experiments in BMSCs. When PMAIP1 was knocked down, there was a suppression in osteogenesis, cell proliferation, and migration of the cells. This further confirmed that PMAIP1 can delay the progression of osteoporosis and serves as a critical molecule for its treatment and prevention.

## CONCLUSIONS

In conclusion, the current research was the first to indicate PMAIP1 as a novel biomarker for the diagnosis of osteoporosis. In addition, based on bioinformatic network analysis and relevant experiments, PMAIP1 may be a crucial therapeutic target for osteoporosis.

## Abbreviation

GEO: Gene Expression Omnibus; PMAIP1: Phorbol-12-Myristate-13-Acetate-Induced Protein 1; GSEA: Gene Set Enrichment Analysis; GO: Gene Ontology; KEGG: Kyoto Encyclopedia of Genes and Genomes; DEG: differentially expressed gene; DEM: differential expression miRNA; DEL: differential expression lncRNA; PPI: Protein and protein interaction; BMSC: bone marrow mesenchymal stem cell; OP: Osteoporosis; BMD: Bone mineral density; PBMC: peripheral blood mononuclear cells; PTH: parathyroid hormone; MTD: mitochondrial targeting domain.

## Supplementary Material

Supplementary Figure 1

Supplementary Table 1
