# Supplementary figures and images for: PMAIP1, a novel diagnostic and potential therapeutic biomarker in osteoporosis
Source: Aging (Albany NY). 2024 Feb 16;16(4):3694–715. doi: 10.18632/aging.205553 (PMC10929792; doi:10.18632/aging.205553)

SUPPLEMENTARY FIGURE

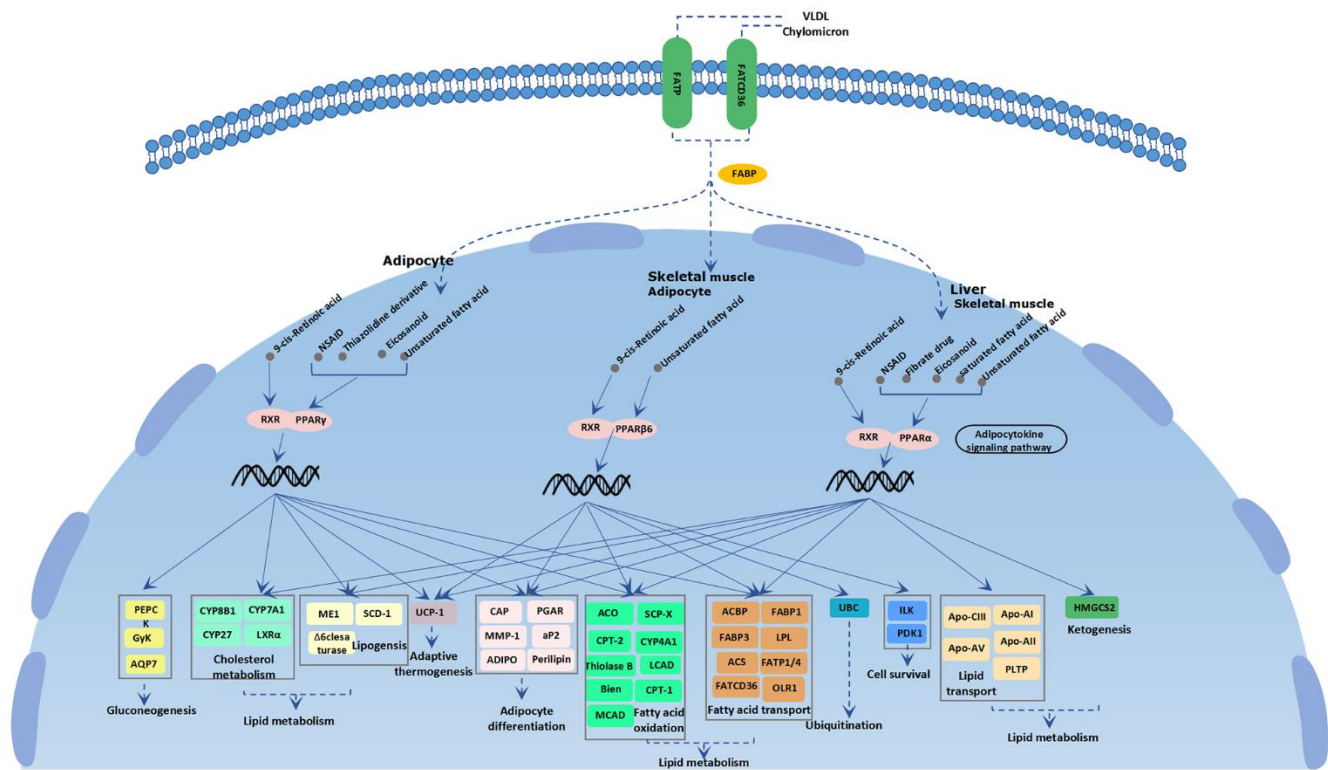

Supplementary Figure 1. PPAR signaling pathway.

Supplement: Supplementary Figure 1 [file aging-16-205553-s001.pdf]
