# Supplementary material for: PMAIP1, a novel diagnostic and potential therapeutic biomarker in osteoporosis
Source: Aging (Albany NY). 2024 Feb 16;16(4):3694–715. doi: 10.18632/aging.205553 (PMC10929792; doi:10.18632/aging.205553)
Supplement: Supplementary Table 1 [file aging-16-205553-s002.pdf]

SUPPLEMENTARY TABLE

Supplementary Table 1. Primers used in this study.

		Primers for qPCR
PMAIP1	F	CGGAGCTGGAAGTCGAGTGT
	R	TTCCTGAGCAGAAGAGTTTGGATA
H1FX-AS1	F	TTGCCGCTGATGTTCCCA
	R	GTTCCCGCCATCATTTCCC
AC009501.4	F	AAGGGCTTGTTTCTGGCTATGTT
	R	GTGGTTGCGTTTCACTGTAAGGA
RP11-5P4.2	F	TCAGGAAGTCAGCCATGCTAAAC
	R	CTTGCCTGCTCACGATTGTTTC
RP4-607I7.1	F	CCTACTCATGTGAGGAAACCAGC
	R	AGTCCTGGGCATGGGAAGTAG
RP5-857K21.4006	F	TCAACAGGAGGAGATAAGGAAGCT
	R	CAGCTCAGGAAGATGACTCAGGG
miR-200b-3p	F	ACACTCCAGCTGGGTAATACTGCCTGGTAA
	R	TGGTGTCGTGGAGTCG
miR-624-3p	F	ACACTCCAGCTGGGCACAAGGTATTGGTA
	R	TGGTGTCGTGGAGTCG
OCN	F	CACTCCTCGCCCTATTGGC
	R	CCCTCCTGCTTGGACACAAAG
OPN	F	CTCCATTGACTCGAACGACTC
	R	CAGGTCTGCGAAACTTCTTAGAT
RUNX2	F	TGGTTACTGTCATGGCGGGTA
	R	TCTCAGATCGTTGAACCTTGCTA
GAPDH	F	GGAAGCTTGTCATCAATGGAAATC
	R	TGATGACCCTTTTGGCTCCC
U6	F	CTCGCTTCGGCAGCACA
	R	AACGCTTCACGAATTTGCGT
